# Predicting near-term glaucoma progression: An artificial intelligence approach using clinical free-text notes and data from electronic health records

**DOI:** 10.3389/fmed.2023.1157016

**Published:** 2023-04-13

**Authors:** Sunil K. Jalamangala Shivananjaiah, Sneha Kumari, Iyad Majid, Sophia Y. Wang

**Affiliations:** Department of Ophthalmology, Byers Eye Institute, Stanford University, Palo Alto, CA, United States

**Keywords:** artificial intelligence, glaucoma, electronic health records, natural language processing, explainability, glaucoma surgery

## Abstract

**Purpose:**

The purpose of this study was to develop a model to predict whether or not glaucoma will progress to the point of requiring surgery within the following year, using data from electronic health records (EHRs), including both structured data and free-text progress notes.

**Methods:**

A cohort of adult glaucoma patients was identified from the EHR at Stanford University between 2008 and 2020, with data including free-text clinical notes, demographics, diagnosis codes, prior surgeries, and clinical information, including intraocular pressure, visual acuity, and central corneal thickness. Words from patients’ notes were mapped to ophthalmology domain-specific neural word embeddings. Word embeddings and structured clinical data were combined as inputs to deep learning models to predict whether a patient would undergo glaucoma surgery in the following 12 months using the previous 4-12 months of clinical data. We also evaluated models using only structured data inputs (regression-, tree-, and deep-learning-based models) and models using only text inputs.

**Results:**

Of the 3,469 glaucoma patients included in our cohort, 26% underwent surgery. The baseline penalized logistic regression model achieved an area under the receiver operating curve (AUC) of 0.873 and F1 score of 0.750, compared with the best tree-based model (random forest, AUC 0.876; F1 0.746), the deep learning structured features model (AUC 0.885; F1 0.757), the deep learning clinical free-text features model (AUC 0.767; F1 0.536), and the deep learning model with both the structured clinical features and free-text features (AUC 0.899; F1 0.745).

**Discussion:**

Fusion models combining text and EHR structured data successfully and accurately predicted glaucoma progression to surgery. Future research incorporating imaging data could further optimize this predictive approach and be translated into clinical decision support tools.

## 1. Introduction

Glaucoma is a chronic progressive disease of the optic nerve and is one of the leading causes of irreversible blindness (1). Many patients remain at an early asymptomatic stage for long periods, while others progress to vision loss and require surgery (2). Although some factors contributing to progression are relatively easy to identify and measure, such as elevated intraocular pressure (IOP) or decreased central corneal thickness, many other factors, such as medication adherence, are less easily characterized (3). It is often difficult for doctors to predict whose glaucoma will worsen. However, now that the digitization of health records has created vast collections of information about patients (including medication and diagnosis information, demographic information, and free-text clinical notes), artificial intelligence (AI) techniques can be developed to analyze patient records and predict ophthalmic outcomes, including glaucoma progression.

Previous efforts have been undertaken to build machine-learning and deep-learning classification algorithms to predict glaucoma progression (4). Many studies have focused on structured information from electronic health records (5–7). Our previous studies have also explored methods for incorporating clinical free-text progress notes into AI prediction algorithms using natural language processing techniques (8, 9). A common challenge has been that these efforts typically have not considered the temporal element of prediction, as most AI prediction algorithms are simple classification algorithms with no specific time horizon. An outcome prediction is most useful when attached to a specific time horizon so that appropriate clinical steps can be taken. Similarly, algorithms trained only on baseline (presenting) information can only be used in limited circumstances and only for new patients. Algorithms that can be deployed at any point in a patient’s clinical course would have broader utility.

The present study aims to develop artificial intelligence models that can predict glaucoma progression to the point of requiring surgery within 1 year, using inputs from electronic health records (EHRs) that are both structured and free-text. The present models would thus be able to be used on glaucoma patients at any time during their treatment course, overcoming a key limitation of previous work. Furthermore, unlike previous models, these models would incorporate temporal information by providing predictions over a fixed time horizon. We compared 3 types of models: a model incorporating information from clinical notes (clinical free text), models using only structured data inputs, and a multimodal fusion model that used both clinical free text and structured data as inputs.

## 2. Methods

### 2.1. Study population and cohort construction:

The overall objective of our algorithm was to predict whether a patient with glaucoma will require surgery within 12 months following a designated encounter visit, given at least 4 months of medical history prior to the encounter date. We narrowed the timeframe under consideration to the near-term future because, although a patient might have surgery at any time in the future (including many years after the initial glaucoma diagnosis), the most relevant prediction is whether the patient will need surgery within the next year. Thus, we carefully constructed a cohort to suit these prediction needs.

We first identified, from the Stanford Research Repository (10), all encounters for patients seen by the Department of Ophthalmology at Stanford University since 2008. We included all patients with at least two encounters with a glaucoma-related diagnosis as determined by the International Classification of Disease Codes (ICD10: H40, H42, or Q15.0; not including glaucoma suspect codes starting with H40.0 and ICD9 equivalents). Theoretically, a model could perform a prediction at any date in a patient’s treatment timeline; for the purposes of our model training, we defined a single prediction date for each patient, on which the model predicts whether that patient would progress to glaucoma surgery within 12 months of that defined date.

**Figure 1:**
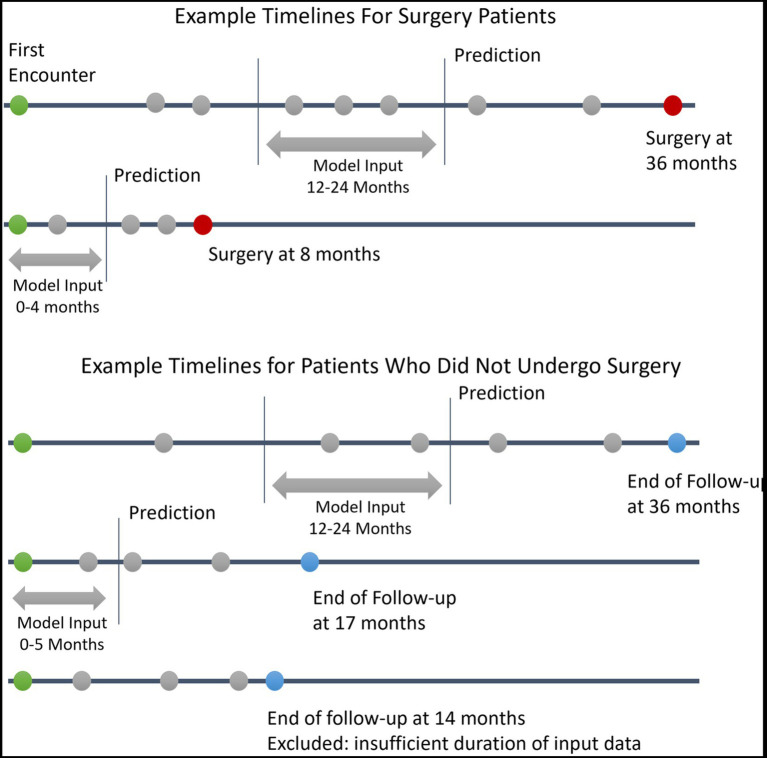
Example Patient Electronic Health Records Timelines. This figure depicts example timelines of patients who did and did not progress to glaucoma surgery. Green circles represent the first patient encounter. Red circles represent surgery dates. Blue circles represent the last follow-up encounter for patients who did undergo surgery. Grey circles represent other encounters with associated clinical data in the electronic health record.

This prediction date effectively divides the patient’s information into a historical look-back period and a future look-forward period. We required a minimum of 4 months and up to 12 months of look-back period during which the clinical information used for the prediction was gathered. The model then predicts whether the patient will progress to require surgery over the following 12 months of look-forward period. Patients without at least 4 months of look-back data or without any clinical progress notes within the look-back period were excluded. For patients who underwent surgery, the prediction date was defined as either 12 months prior to surgery or after the initial 4 months of follow-up (whichever was later). For patients who did not undergo surgery, a minimum of 12 months of follow-up after the initial lookback period was required to ascertain that no surgery was performed over the entire look-forward period; the prediction date was defined as 12 months prior to the last follow-up visit, with the caveat that only patients with at least 4 months of historical lookback were included. A summary of cohort construction timelines with example patients is given in Figure 1. This formulation of the cohort and the prediction date is similar to that used in a previous study predicting near-term palliative care needs among inpatients (11).

**Figure 2:**
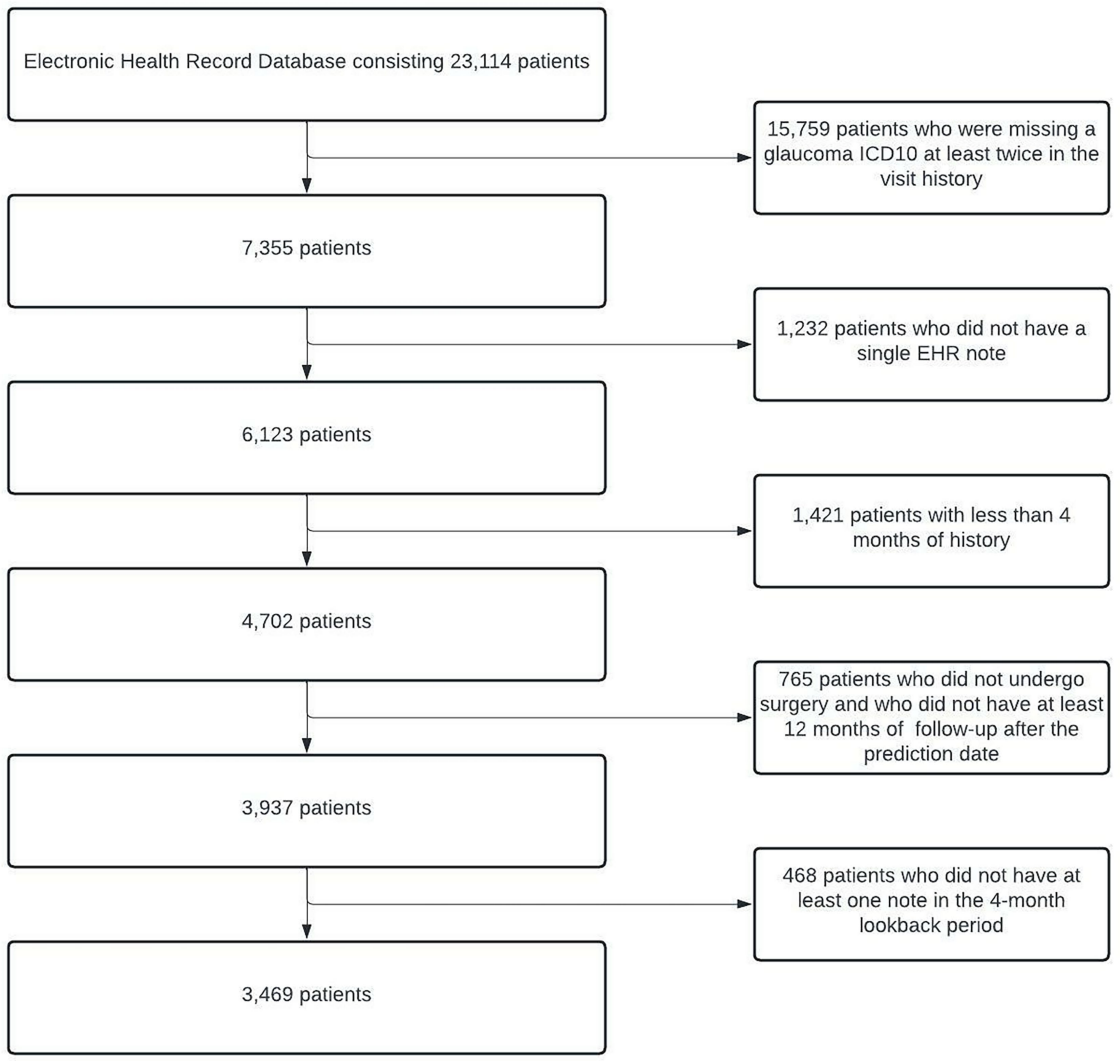
Cohort Description and Construction. Flowchart depicting the process of identifying eligible glaucoma patients from EHRs. At the end of all of the processing steps, 3,469 patients were included in the study. EHRs, electronic health records.

With our inclusion/exclusion criteria, the final cohort included 3,469 patients. A cohort identification flow diagram is shown in Figure 2. We randomly divided our cohort into training, validation, and test groups in 70% (*N* = 2,429), 15% (*N* = 520), and 15% (*N* = 520) proportions, respectively. The proportion of patients who progressed to surgery in each of these groups was 25.9% (*N* = 629), 26.0% (*N* = 135), and 26.9% (*N* = 140), respectively. The validation set was used for model and hyperparameter tuning, and the final evaluation was performed on the test set.

### 2.2. Feature engineering

#### 2.2.1. Text data preprocessing

Ophthalmology clinical progress notes from the look-back period were identified and concatenated such that the most recent notes appeared first. All notes were lower-cased and tokenized. Punctuation and stop words [Nltk library (12)] were removed. We mapped each word of the document with previously trained 300-dimensional ophthalmology domain-specific neural word embeddings (13) for input into models. To understand the general characteristics of the words associated with patients who progressed to surgery and patients who did not, we calculated pointwise mutual information (14) for words that occurred in the notes of at least 20 different patients.

#### 2.2.2. Structured data preprocessing

Information on the patient’s demographics (age, gender, race/ethnicity), diagnoses (International Classification of Disease codes), medications, and eye examination results (visual acuity, intraocular pressure, and central corneal thickness of both eyes) was obtained from each patient’s look-back period. Visual acuity was converted to mean logarithm of the minimum angle of resolution (logMAR). Numeric data were standardized to a mean of 0 and standard deviation of 1. We identified the low, medium, high, and most recent values for each eye examination feature. For the medication and diagnosis data, we filtered out features with <1% variance. For missing values in numeric features, we created a missing indicator column for the feature after performing column mean imputation. Categorical variables were converted to a series of Boolean dummy variables. After the final preprocessing, 127 structured features remained.

### 2.3. Modeling

#### 2.3.1. Text model

**Figure 3:**
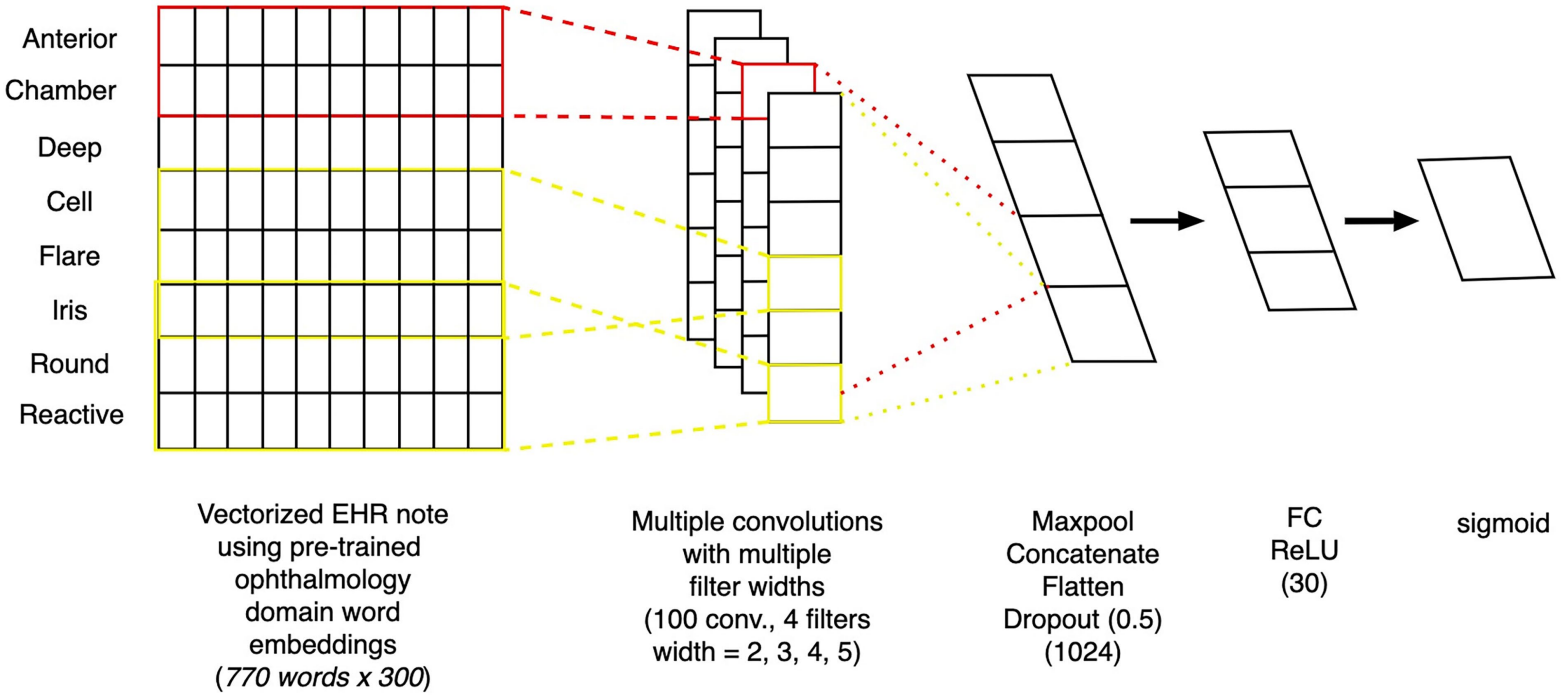
Architecture of convolutional neural network text model. Depiction of the architecture of a convolutional neural network model, which takes as inputs free-text from clinical notes and outputs the probability of whether the patient will progress to surgery within 12 months.

To create a model that uses free text from clinical notes as the input, we built a one-dimensional convolutional network model (15), a similar style of which was previously been demonstrated to work well on ophthalmology notes (9). Figure 3 depicts the architecture of the text model. The free-text clinical notes were input into the model after padded or truncation to a length of 770 tokens (the 80th percentile length of notes). We applied a set of 25 one-dimensional convolutional filters, each of sizes 2, 3, 4, and 5, followed by max pooling. The outputs of these filters were concatenated and passed through 2 additional fully-connected layers with dropout to obtain our final prediction. For training, we used the Adam optimization algorithm. We tuned the model’s architecture, learning rate (0.0003), weight decay factor (0.01), and batch size (32) to optimize validation loss.

#### 2.3.2. Structured EHR data model

To create a model that uses structured EHR data as the input, we started by building a basic L1 penalized logistic regression model with the structured data. We also trained several tree-based models, including random forest and extreme gradient boosting (XGboost), and saw that these models performed better on our dataset than our baseline model. We tuned the maximum depth, minimum samples per leaf node, and the number of trees for the tree-based models. We then built a fully connected neural network model based on the structured data, as follows: The input features were fed into the neural network with 2 hidden layers of 60 and 30 nodes. The first hidden layer used an input of 60 nodes, followed by ReLU activation and a dropout layer with a probability of 0.5. The second hidden layer had 30 nodes, also followed by ReLU activation and a dropout layer with a probability of 0.5. Then, a final prediction layer used softmax activation for classification. Models’ hyperparameters were tuned using 5-fold cross validation (Table 1).

**Table 1:** Hyperparameters for the tuned models.

Modeling method	Data	Hyperparameters
Logistic regression	Structured data	Penalty: L1
		Max iteration: 100
		Scoring: roc_auc
XGboost	Structured data	N_estimators: 100
		Max depth: 3
		Learning rate: 0.1
		Reg_lambda: 0
Gradient boosted trees	Structured data	Learning rate: 0.1
		Max depth: 3
		N_estimators: 100
		Subsample: 0.5
Random forest	Structured data	Max depth: 50
		Min samples per leaf node: 30
		N_estimators: 1200
Deep learning	Structured data	Learning rate: 0.0005
		Weight Decay: 0.1
		Batch Size: 32

#### 2.3.3. Multimodal fusion model

**Figure 4:**
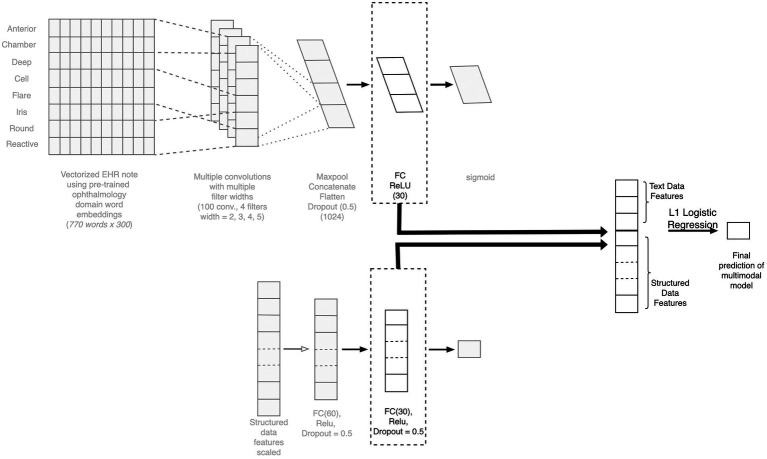
Architecture of the fusion model combining text and structured data. Depiction of the architecture of our multimodal fusion model, which fuses both structured and free-text modalities of data. The final layers of the TextCNN is concatenated with the final layer of the structured model neural network to create a combined feature vector for final prediction. CNN, competitive neural network; EHR, electronic health record; FC, fully connected.

To create a model that considers both clinical free text and structured EHR data as the input, we created a neural network-based multimodal model. Each layer of the neural network can be thought of as feature engineering, wherein the model is automatically engineering these layers. We extracted the final layer of the text model and the deep learning-based structured model (as these are features curated by the individual models), combined the features, and then applied L1 penalized logistic regression to predict the outcome. The model architecture is depicted in Figure 4.

### 2.4. Evaluation

We evaluated the performance of all our models on the held-out test dataset (data set aside for testing the model after training and validation) using precision (also known as positive predictive value), recall (also known as sensitivity), F1 score (the harmonic mean of the precision and recall), the area under the receiver operating curve (AUROC), and the area under the precision-recall curve (AUPRC). For metrics of precision, recall, and F1 score, which are reported at a single classification threshold, the optimal threshold was tuned on the validation set for the best F1 score; the final precision, recall, and F1 scores were evaluated on the test set using this optimized threshold.

### 2.5. Explainability

#### 2.5.1. Explainability for the structured features

To understand which structured features had more predictive power, we used SHapley Additive exPlanations (SHAP) values (16). This technique calculates the importance of the features based on the magnitude of feature attributions, using a game theory approach to explain the results of any machine learning model and make them interpretable by measuring the feature contribution to individual predictions. We used SHAP TreeExplainer (17), which estimates the SHAP values for tree-and ensemble-based models, on the best random-forest model.

#### 2.5.2. Explainability for the text model

GradCAM, a class-discriminative localization technique originally proposed by Selvaraju et al. (18) that generates visual explanations for any convolutional neural network (CNN) without requiring architectural changes or re-training. The technique was further adapted for Text-CNN (19). We used this technique to investigate the key phrases that led our Text-CNN model to predict that a given patient would require surgery within 12 months.

## 3. Results

### 3.1. Population characteristics:

Population characteristics of the subjects included in the study are summarized in Table 2. Of these patients, 26% went on to require glaucoma surgery. The mean age of the patients in the study was 67 years, and the mean IOP for both eyes was close to 15 mmHg. The mean logarithm of the minimum angle of resolution visual acuity for both eyes (mean logMAR) was around 0.55 for the right eye and 0.65 for the left eye (Snellen equivalent approximately 20/70 and 20/90, respectively). The population in the study was predominantly Asian and White. To gain an overview of words most highly associated with patients who progressed to surgery and those who did not, pointwise mutual information was calculated for words that occurred in at least 20 patients. The top 10 words with the highest pointwise mutual information scores are presented in Supplementary Table S1.

**Table 2:** Population characteristics.

Characteristics	Total (*n* = 3,469)	No surgery (*n* = 2,565)	Surgery (*n* = 904)
Age (years)	67 ± 18	69 ± 17	66 ± 18
Intraocular pressure (mmHg)			
Right eye	15.4 ± 6.1	15.1 ± 5.0	15.5 ± 6.8
Left eye	15.4 ± 6.2	15.3 ± 5.7	15.4 ± 6.6
Visual acuity (logMAR)			
Right eye	0.55 ± 0.84	0.48 ± 0.81	0.61 ± 0.86
Left eye	0.65 ± 0.92	0.59 ± 0.93	0.70 ± 0.92
Sex			
Female sex	1766 (50.9)	1,330 (51.8)	436 (48.2)
Male sex	1703(49.1)	1,235 (48.2)	468 (51.8)
Race and ethnicity			
Asian	997 (28.7)	707 (27.5)	290 (32.1)
White	1,444 (41.6)	1,138 (44.4)	306 (33.8)
Hispanic	378 (10.9)	251 (9.8)	127 (14.0)
Black	142 (4.2)	98 (3.8)	44 (4.9)
Other	508 (14.6)	371 (14.5)	137 (15.2)

logMAR = logarithm of the minimum angle of resolution. Data are presented as mean ± standard deviation or number (%). Numeric variables are reported here as conventionally seen, for ease of interpretation. They were standardized to a mean of 0 and variance of 1 before being applied to the modeling approaches.

### 3.2. Model results

**Figure 5:**
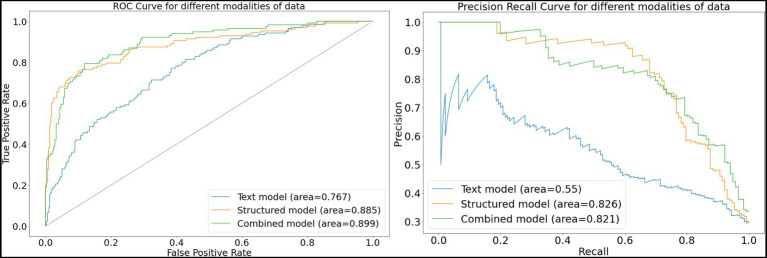
Receiver-operating and precision-recall curves for models. This figure depicts receiver operating characteristic curves and precision recall curves for models predicting glaucoma progression to surgery. The free-text model uses only clinical notes as inputs into a convolutional neural network; the structured model uses only structured electronic health records data as inputs into a deep learning model; and the combined model fuses both structured and text inputs into a fusion deep learning model.

For the structured data models, the L1 penalized logistic regression model resulted in an AUC score of 0.873 and F1 score of 0.750. Tree-based models resulted in AUC and F1 of 0.870 and 0.757 (XGboost), 0.871 and 0.749 (gradient boosted trees), and 0.876 and 0.746 (random forest). The deep learning structured model resulted in AUC of 0.885 and F1 score of 0.757. For all models, the classification threshold was tuned to optimize F1 score on the validation set. Receiver operating characteristic curves and precision-recall curves for the best structured, text, and combined deep learning models are shown in Figure 5. The combined model, which included both structured and free-text features, outperformed the structured-data-only model and free-text-only model. The free-text-only model resulted in an AUC of 0.767, and the combined model had an AUC of 0.899. Table 3 presents the F1 score and the corresponding precision and recall scores for each of the modalities of data based on deep learning models.

**Table 3:** Performance metrics.

Modality	Precision	Recall	F1 score	Threshold
Text model	0.3959	0.8285	0.5357	0.4
Structured model	0.8000	0.7187	0.7572	0.55
Multimodal model	0.7022	0.7931	0.7449	0.35

### 3.3. Explainability

#### 3.3.1. Explainability for structured data

**Figure 6:**
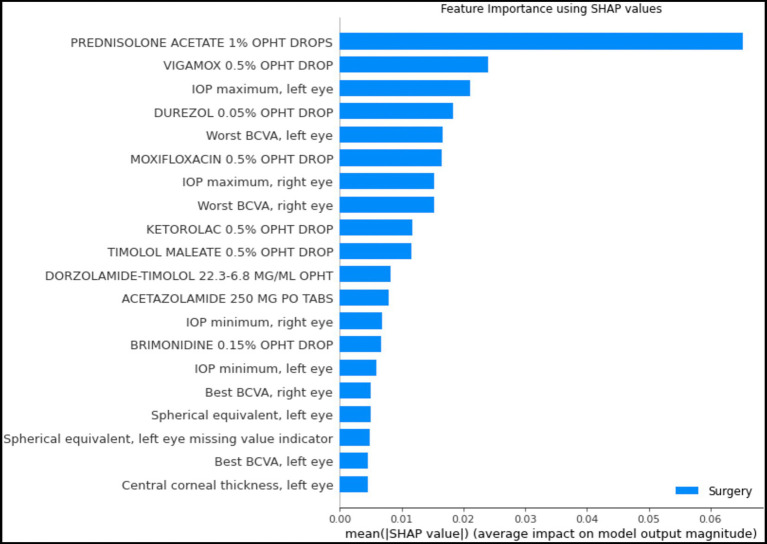
Shapley feature importance for structured data. The figure depicts the mean absolute Shapley value for the top 20 most important features in the structured data for predicting whether a patient would progress to the point of requiring surgery within the next year, using the random forest model. The mean absolute Shapley value was calculated for all patients in the test set.

Figure 6 depicts the mean absolute Shapley values for the top 20 most important features from the structured data for predicting which patients will require surgery, using the random forest model. The most important features include the use or nonuse of glaucoma medications as per the medication lists, IOP, VA, and refraction spherical equivalent. These features are similar to the factors considered by glaucoma specialists when they predict a glaucoma patient’s prognosis with respect to the need for surgery.

#### 3.3.2. Explainability for text data

**Figure 7:**
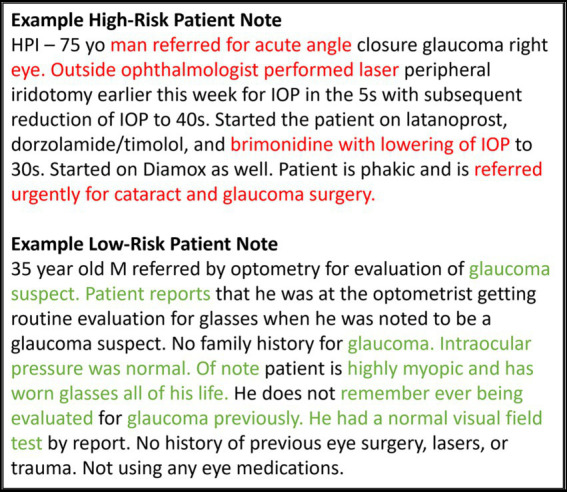
Feature importance for text data. This figure shows example notes for glaucoma patients at high and low risk for progressing to glaucoma surgery. The GRAD-CAM-Text method was used to identify words important to the model prediction, highlighted in red for predicting surgery and in green for predicting no surgery.

Figure 7 highlights words and phrases in example clinical progress notes that were identified by GradCAM-text as most important for model predictions. For a high-risk patient, these explainability methods highlight clinical features that tend to indicate acute risk of surgery (“Outside ophthalmologist performed laser,” “referred urgently for cataract and glaucoma surgery” etc.), while for a low-risk patients, highlighted clinical features are generic or low risk (“glaucoma suspect,” “intraocular pressure was normal” etc.), which is in alignment with the expectations of glaucoma specialists.

## 4. Discussion

In the present study, we developed an AI approach that successfully predicts whether glaucoma patients would require surgery in the following 12 months based on EHR structured data and clinical progress notes. The study compared the results from 3 different approaches: (1) a deep-learning model which used doctor’s free-text progress notes as input; (2) traditional machine-learning and deep-learning modeling approaches, which used structured EHR data as input; and (3) fusion deep-learning models, which used both structured EHR data and free-text notes as input. The resulting predictions indicated that fusion models trained using both structured EHR data and free-text notes as features performed better than models using either structured EHR only or free-text notes only. Explainability studies showed that models relied upon clinically relevant features in both the structured and text inputs.

Our work expands upon previous work which has generally focused on using single modalities of data. Baxter et al. explored many deep-learning and tree-based models for structured EHR data inputs but concluded that a logistic regression model had the best performance, with an AUC of 0.67 (4). Hu et al. demonstrated that using massively pre-trained language models for clinical free-text notes could improve the AUC to 0.70 (20). Wang et al. achieved an AUC of 0.73 on structured data only and an AUC of 0.70 using text features only. Using our combined model to predict glaucoma surgery in the near term, we were able to achieve an AUC of 0.899.

In addition to achieving significantly higher AUC, our model has inherent flexibility in terms of input that is in line with the needs of real-life patients and physicians in the clinic. Previous studies were limited to predicting a patient’s prognosis regarding the need for surgery using data from their initial visit or data included from the baseline period. Some studies focused on predicting future surgery over all time, which sometimes meant predicting surgery even 10 years into the future (9, 20). A unique strength of our study was the formulation of a model that could be used for any glaucoma patient at any point during their follow-up, rather than just at their initial visit or within a restricted baseline period, to predict the dynamic probability of whether the patient will require a surgical procedure within 1 year from the prediction date. Furthermore, considering more recent or updated information in the present models likely caused the prediction performance to improve.

We also investigated what types of information models were relying upon for prediction to improve transparency and trustworthiness for these AI models, a common criticism of which is that they are “black boxes” difficult for clinicians to understand. For the free-text model, explainability studies using GradCAM-Text showed the key phrases from the notes that were most important for the predictions, which were aligned with clinical expectations: for example, “referred urgently.” Similarly, for the structured-EHR-data models, analysis of Shapley values showed the most important features included the use of various glaucoma medications, intraocular pressure, refraction, and visual acuity. These are similar features to those clinicians would take into consideration when making a decision (2).

This study has several remaining limitations and challenges. Our models are built and validated on patients who visited a single academic center, which may limit generalizability. Additionally, the input text length was limited, which is a challenge of deep learning architectures for incorporating text. To develop a model that can predict prognosis for any patient at any point in their treatment trajectory, it must be taken into consideration that every patient will have different amounts, and differing complexity, of data as input. Structured data can be easily summarized (e.g., most recent measurement values, highs, lows, medians, or even presence of specific conditions) but raw inputs of text require every word to be input, and models must have a standardized input length. To solve this problem, some sort of meaningful summary representation of a variable amount of text history would be required, to reduce the text to a standardized input size. Furthermore, although we could perform explainability studies for the text and structured data models, there are no commonly used methods to investigate explainability in the multimodal models, which could be an area for future research.

In conclusion, we used multimodal electronic health records data to develop models to predict which glaucoma patients were likely to progress to surgery in the following 12 months, significantly outperforming previous models built for similar tasks. We showed that both text-based and structured-data-based models relied upon clinically relevant information to make predictions. Fusion models relying on both structured data and text notes, while lacking explainability, may improve model performance as compared with models relying on only structured data and only free-text notes. To take the next step towards translation into clinical decision support tools, further research is needed to improve explainability and performance, potentially by incorporating larger data sources and imaging data modalities.

## Data availability statement

The data analyzed in this study is subject to the following licenses/restrictions: The dataset contain protected health information of individuals and cannot be publicly shared; for those wishing to collaborate, please contact the corresponding author. Requests to access these datasets should be directed to SW, sywang@stanford.edu.

## Ethics statement

The studies involving human participants were reviewed and approved by Stanford University Institutional Review Board. Written informed consent for participation was not required for this study in accordance with the national legislation and the institutional requirements.

## Author contributions

SJ, SK, and SW contributed to data analysis. SW contributed to data acquisition and supervised the study. All authors contributed to the interpretation of the data, drafting and critical revision of the manuscript, and approval of the final manuscript.

## Funding

This work was supported by National Eye Institute K23EY03263501 (SW); Career Development Award from Research to Prevent Blindness (SW); unrestricted departmental grant from Research to Prevent Blindness; and departmental grant National Eye Institute P30-EY026877.

## Conflict of interest

The authors declare that the research was conducted in the absence of any commercial or financial relationships that could be construed as a potential conflict of interest.

## Publisher’s note

All claims expressed in this article are solely those of the authors and do not necessarily represent those of their affiliated organizations, or those of the publisher, the editors and the reviewers. Any product that may be evaluated in this article, or claim that may be made by its manufacturer, is not guaranteed or endorsed by the publisher.
