# Supplementary material for: Predicting near-term glaucoma progression: An artificial intelligence approach using clinical free-text notes and data from electronic health records
Source: Front Med (Lausanne). 2023 Apr 13;10:1157016. doi: 10.3389/fmed.2023.1157016 (PMC10133544; doi:10.3389/fmed.2023.1157016)
Supplement: Supplementary file 1 [file Data_Sheet_1.PDF]

Supplementary Table 1. Words most associated with notes of patients who progressed to surgery and those who did not

Rank	Surgery Notes	Pointwise Mutual Information	No Surgery Notes	Pointwise Mutual Information
1	Neptazane	1.03	pericentral	0.30
2	bscan	0.84	prosthesis	0.26
3	coverage	0.79	antioxidant	0.26
4	seidel	0.77	serial	0.24
5	methazolamide	0.76	reconstructive	0.24
6	phacod	0.76	supplementation	0.24
7	baerveldt	0.76	lube	0.23
8	nylon	0.75	compression	0.23
9	yachna	0.75	lubrication	0.23
10	canal	0.73	assurance	0.22
